# Perceptions of individual and societal onset of old age: associations with views on aging in a sample aged 16 to 96 years

**DOI:** 10.1007/s10433-025-00886-6

**Published:** 2025-09-23

**Authors:** Markus Wettstein, Anna E. Kornadt, Lisa Marie Warner, Eva-Marie Kessler

**Affiliations:** 1 Department of Psychology, Developmental Psychology, Humboldt-Universität Zu Berlin, Rudower Chaussee 18, 12489 Berlin, Germany; 2 Department of Prevention Research and Social Medicine, Institute for Community Medicine, University Medicine, Greifswald, Germany; 3 Department of Behavioral and Cognitive Sciences, University of Luxembourg, Esch–sur–Alzette, Luxembourg; 4 Department of Psychology, MSB Medical School Berlin, Berlin, Germany

**Keywords:** Age threshold, Age categorization, Subjective age views, Subjective age, Ageism, Age stereotypes

## Abstract

There are considerable interindividual differences regarding when individuals perceive someone as “old” (i.e., perceived individual onset of old age). Individuals might also differ in when they believe that society considers someone as “old” (i.e., perceived societal onset of old age). We investigated how multiple indicators of views on aging (age stereotypes, subjective age, age knowledge, perceived ageism), socio-demographic factors (age, sex, education, region of residence), and self-rated health are related to perceptions of individual vs. societal onset of old age and with the difference between both measures in an age-heterogeneous sample. In the Age_ISM Germany survey, a representative sample of 2,000 Germans was recruited (age range 16–96 years, *M* = 56.6 years). We ran structural equation models with sampling weights and found that individuals report a perceived individual onset of old age that was on average more than eight years later than their perceived societal onset of old age. Perceived ageism was associated with an earlier perceived individual and societal onset of old age as well as with a greater discrepancy between both indicators. Feeling younger was associated with a later perceived individual onset of old age. Associations of views on aging, socio-demographics, and self-rated health with perceived individual onset of old age did not vary across age groups, whereas age-group differences emerged for perceived societal onset of old age. Our findings advance theoretical frameworks on views on aging by demonstrating a meaningful discrepancy between perceived individual and societal onset of old age, which are uniquely associated with views on aging.

Individuals have a concept of when old age begins (Barrett & Von Rohr 2008; Chopik et al. 2018; Wettstein et al. 2024). While different terminologies coexist, we refer to this concept as “perceived onset of old age” (Barrett & Von Rohr 2008; Wettstein et al. 2024), which comprises both individual perceptions of when old age begins (i.e., perceived individual onset of old age) and perceptions of when society considers someone as old (i.e., perceived societal onset of old age). Perceived onset of old age is based on scripts that “help to organize our lives and reduce uncertainty about the future” (Billari et al. 2021). When individuals perceive old age to begin does not only tell us how individuals understand and structure their life span but may also have important implications for when—or if at all—they start preparing for aging. Moreover, an earlier perceived onset of old age (or age of leaving middle age) has been shown to be associated with poorer subsequent physical and mental health (Kuper and Marmot 2003).

So far, little is known about psychological factors that may shape individuals’ perceptions of when old age begins. While it is plausible that views on aging, i.e., how individuals perceive and evaluate their own aging, including how old they feel, and how they perceive older adults in general (Hess 2006; Shrira et al. 2022; Westerhof & Wurm 2015), are associated with the perceived onset of old age, empirical evidence to date is sparse. Further, socio-demographic indicators and views on aging may be differentially related to the perceived individual onset of old age versus the perceived societal onset of old age.

The aim of the present study is to investigate how the onset of old age is associated with of views on aging, socio-demographic, and health-related factors. Diehl and Wahl (2024) suggest a theoretical framework for the study of self-perceptions of aging and distinguish between (1) distal antecedents, including socio-demographic factors (age, sex, education), biological/health-related factors, and psychological factors, and (2) proximal antecedents, including experiences of age stereotyping. We build on this framework by considering the perceived onset of old age as outcome and by including socio-demographic factors (age, sex, education, region of residence), health-related factors (self-rated health), and psychological factors (knowledge about old age and older adults) as distal factors associated with perceived onset of old age. In addition, we include the proximal factor experiences of age stereotyping, operationalized via perceived ageism as well as the endorsement of age stereotypes and subjective age (see Fig. 1). We go beyond previous studies by (1) investigating both perceived individual onset of old age and perceived societal onset of old age, as well as the difference between both onset measures, (2) using data from an age-heterogeneous sample ranging from adolescence to very old age, and (3) by including a broad set of factors related to perceived onset of old age, such as age stereotypes or perceived ageism, whose unique and independent associations with perceived onset of old age have rarely been empirically examined. We will also explore whether these associations vary according to chronological age.

**Fig. 1:**
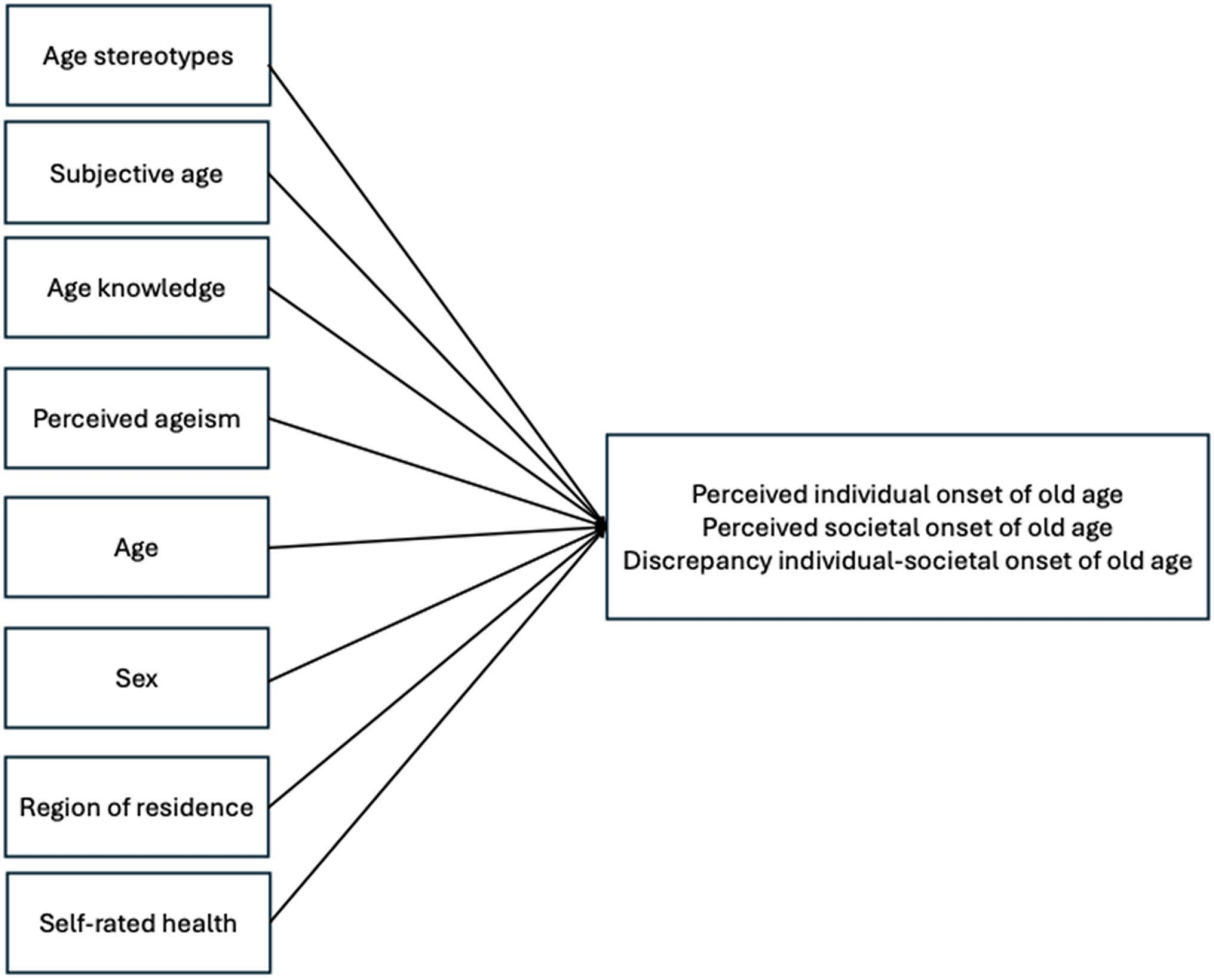
Overview of the conceptual model. Various views on aging domains (age stereotypes, subjective age, age knowledge, perceived ageism) as well as socio-demographic characteristics (age, sex, region of residence) and self-rated health are assumed to be systematically related to perceived individual and societal onset of old age as well as to the discrepancy between perceived individual onset of old age and perceived societal onset of old age

## Perceived onset of old age and views on aging

Views on aging refer to how individuals perceive and evaluate aging and older adults as well as their own aging (Hess 2006; Westerhof & Wurm 2015). They are multidimensional (Kornadt et al. 2018; Shrira et al. 2022) and comprise various domains, including, for instance, age stereotypes and subjective age.

### Age stereotypes

One key construct within the umbrella of views on aging is (descriptive) age stereotypes, which are beliefs and cognitions about older people and old age in general (Kornadt & Rothermund 2011). As age stereotypes are domain-specific and multi-faceted (Kornadt & Rothermund, 2011), we investigate different age stereotype domains (e.g., stereotypes about family and partnership or about work and employment of older adults; see Kornadt & Rothermund 2011) rather than “general” age stereotypes that do not allow for such a differentiation. The only available evidence on the association between age stereotypes and the perceived onset of old age shows that individuals who believed that old age begins at a certain chronological age—as opposed to those who did not assign a certain chronological age to the beginning of old age—, and particularly those who believed that old age begins at a younger chronological age, held more stereotypes about old people (Tuckman & Lorge 1953). However, this evidence dates back more than seven decades. Nowadays, people with negative age stereotypes may have these negative stereotypes because they associate “old age” with very old adults who are limited in their health and relatively close to death and less so with the “young-old” adults, who tend to have better functioning and more psychosocial resources than the “old-old” (Baltes & Smith 2003). In such a scenario, more negative age stereotypes should be associated with a later perceived onset of old age. This association also aligns with an “age-group dissociation” point of view (Weiss & Freund 2012; Weiss & Kornadt 2018; Weiss & Lang 2012), with those who hold the most negative age stereotypes feeling younger (Kornadt et al., 2023) and potentially placing the onset of old age further away from their own age in order to psychologically distance themselves from the unwanted label of “old age.”

### Age knowledge

Another domain of views on aging which is related to age stereotypes is knowledge about aging and old age (Palmore 1977). In the Age_ISM Germany survey, participants systematically overestimated the prevalence of depressive symptoms among older adults, as well as the proportion of older adults living in nursing homes (Kessler & Warner 2023). Such limited knowledge, reflecting biased and rather negative perceptions of old age, might be associated with negative age stereotypes and might, through the age-group dissociation mechanism (Weiss & Freund 2012; Weiss & Kornadt 2018; Weiss & Lang 2012), contribute to a perceived later onset of old age, whereas those with more accurate knowledge may report an earlier perceived onset of old age.

### Subjective age

Individuals who feel younger set both the perceived end of midlife and the perceived onset of old age later (Toothman & Barrett 2011; Wettstein et al. 2024). For instance, someone who feels “young” at age 60 will probably not believe that old age starts already at age 60, whereas someone who feels “old” at that age may believe that old age has already begun. We therefore suggest that feeling younger is associated with a later perceived onset of old age.

### Perceived ageism

Finally, perceived ageism, i.e., the subjective perception of being looked at in a negative way or treated unfairly due to one’s chronological age (Ludwig et al. 2024), may also shape individual conceptions of the onset of old age. Individuals who report having experienced ageism or age discrimination report feeling older (Stephan et al. 2015); they might thus attribute the experience of ageism to their age and consequently feel older. Therefore, we assume that perceived ageism is related to the perceived onset of old age, with those who report having experienced ageism setting a lower threshold for the onset of old age than those with no such experience.

Taken together, as illustrated in Fig. 1, we hypothesize that all of these views on aging domains—age stereotypes, subjective age, age knowledge, and perceived ageism—are associated with the perceived onset of old age.

## The role of socio-demographic factors

Previous research has found that women perceive old age to start later than men (Ayalon et al. 2014; Barrett & Von Rohr 2008; Chopik et al. 2018; Drevenstedt 1976; Frąckowiak et al. 2020; Toothman & Barrett 2011). This sex difference is potentially due to women’s higher life expectancy (German Federal Statistical Office, n.d.; Eurostat 2022). Another reason could be that age stereotypes targeted at older women are more negative than those targeted at older man (double standard of aging; Sontag 1982). Although a recent meta-analytic review found that attitudes toward older women and men are equivalent (Shakeri & North 2025), the double standard seems to persist in other domains, e.g., in the media, where older women are portrayed more negatively than older men (Bazzini et al. 1997; Lauzen 2021; Lauzen & Dozier 2005). The double standard of aging might explain the stronger tendency of women as compared to men to psychologically distance themselves from old age (“age-group dissociation”; Weiss & Kornadt 2018) by setting an older onset of old age.

Higher levels of education were also found to be associated with a later perceived onset of old age (Ayalon et al. 2014; Kuper & Marmot 2003; Toothman & Barrett 2011), which could be due to the higher life expectancy, better health outcomes (Crimmins & Zhang 2019), as well as better access to resources among those with higher levels of education.

Moreover, differences in perceived onset of old age according to region of residence within Germany were reported (Wettstein et al. 2024), with a later perceived onset of old age in West Germany as compared to East Germany. Whereas health and life expectancy disparities between West and East Germany have become considerably smaller across time (Lampert et al. 2019), “the reunification of West and East Germany in 1990 merged two vastly different systems, resulting in distinctive patterns of inequality that persist today” (Kronauer & Goebel 2025). These two different political systems might thus not only have an enduring impact on socioeconomic inequality, but also on individuals’ values and attitudes, including their perceptions and conceptualizations of old age.

## The role of health

Health also seems to play a role for the perceived onset of old age (Demakakos et al. 2007; Kuper & Marmot 2003); individuals who perceive their health as better report a later perceived onset of old age (Ayalon et al. 2014), and they believe that midlife starts and ends later than do individuals with poorer self-rated health (Kuper & Marmot 2003; Toothman & Barrett 2011). Individuals who feel less healthy might interpret their health constraints as an “aging body reminder” (Barrett & Gumber 2020) and attribute their poor health to their age (Levy et al. 2009, 2023), thus concluding that old age has already begun, whereas older individuals with good self-rated health might feel younger than their age and also believe that the onset of old age is still ahead of them.

## The moderating role of chronological age

Little is known about age differences in associations of the perceived onset of old age with views on aging. From the conceptual perspective of stereotype embodiment theory (Levy 2009), views on aging become more self-relevant and salient as individuals reach old age. For instance, Chopik et al. (2018) discuss that “as people age, they become increasingly closer to identifying with a stigmatized group (i.e., older adults)” (p. 5). Indeed, age stereotypes are self-directed as soon as individuals reach old age—or as soon as they consider themselves “old”. The mechanisms of age-group dissociation described above may thus operate particularly among older adults, whereas younger and middle-aged adults may feel less in need for age-group dissociation in order to distance themselves from older adults due to their perceived distance from old age. Indeed, Rupprecht et al. (2025) found that individuals who are chronologically older set the onset of old age later for domains that they consider as self-relevant. We therefore expect stronger associations between the views on aging indicators and perceived onset of old age in late adulthood (i.e., 60 years and older) as compared to young adulthood and midlife (i.e., 16–59 years), whereas socio-demographic factors and self-rated health might reveal similar associations with perceived onset of old age at different ages.

## The present study

With the exception of very few studies (Chopik et al. 2018; Kessler & Warner 2023; Wurm et al. 2025), prior findings on perceived onset of old age were restricted in their age range, as “most studies examine only one age group’s perceptions of developmental transitions (…) or ignore certain groups (e.g., middle-aged adults) entirely by comparing only younger and older adults” (Chopik et al. 2018, p. 3). Furthermore, while several studies have addressed socio-demographic predictors of the perceived onset of old age, less is known about the association of perceived onset of old age with views on aging. Moreover, whereas the summarized evidence referred to factors associated with the perceived *individual* onset of old age, there is, to our knowledge, no evidence available so far with regard to correlates or determinants of the perceived *societal* onset of old age.

This study uses data from the Age_ISM Germany survey to investigate both the perceived individual and societal onset of old age in a sample with an age range from 16 to 96 years and to investigate associations of various views on aging domains as well as of socio-demographic factors and self-rated health with both onset of old age indicators. Our hypotheses are as follows:

1. Views on aging: feeling younger, having more negative age stereotypes, and having more accurate knowledge about old age is associated with a later perceived onset of old age, whereas reported experiences of ageism are associated with an earlier perceived onset of old age.
2. Socio-demographic factors: Chronologically older individuals, women, individuals in West Germany as well as those with higher levels of education report a later perceived onset of old age.
3. Health: Individuals with a better self-rated health have a later perceived onset of old age than individuals with a poorer self-rated health.
4. Chronological age as a moderator: Among chronologically older adults, the views on aging indicators are more closely associated with perceived onset of old age than among chronologically younger adults, whereas associations of socio-demographic factors and health with perceived onset of old age do not vary by age.

Due to the lack of prior evidence, we investigate in an exploratory way whether the associations of views on aging, socio-demographic factors, and self-rated health with perceived individual onset of old age differ from their associations with perceived societal onset of old age. Furthermore, we also test whether the difference between perceived societal onset of old age and perceived individual onset of old age is related to views on aging, socio-demographic variables, and self-rated health.

## Method

### Sample and procedure

Participants were recruited as part of the project Age_ISM Germany (Kessler & Warner 2023). A nationally representative^1^ sample of the German general population aged 16 and older was drawn.

Data were collected via a CATI (Computer Assisted Telephone Interviewing) dual frame approach; that is, in order to adequately represent both landline users and those without landline access and to avoid selection bias, a landline/mobile phone ratio of 60–40 percent was applied. Potential participants were contacted via randomly allocated landline or mobile telephone numbers. This recruitment procedure allowed the full realization of 2,000 telephone-based interviews. The average overall interview duration, including all measures and interview components, was 23.7 min. Prior to the survey, study participants provided verbal consent. Research procedures and ethics were approved by the institutional ethics commission of MSB Medical School Berlin (#MSB-2021/75).

A sample description is provided in Table 1. Mean age of the sample was 56.56 years (SD = 16.47 years; range 16–96 years). Within the study sample, 50.1% were female.

**Table 1:** Descriptive statistics at baseline assessment

	M	SD
Perceived individual onset of old age (45–95)	71.03	8.43
Perceived societal onset of old age (35–90)	61.73	9.71
Perceived individual minus Perceived societal onset of old Age (− 20–50)	9.27	9.56
Age (16–96)	56.56	16.47
% Women	50.1%	
% East Germany	24.8%	
Education (1–4)^a^	2.76	1.05
Self-rated health (1–5)^b^	2.33	0.88
Age stereotypes (1–4)^c^		
Most older adults find the right solution when coping with important matters	2.89	0.71
Most older people are lonely	2.24	0.76
Most older adults have plenty of money and can spend their money for nice personal experiences	2.14	0.79
Most older adults cannot attune to changes anymore and therefore are inferior to younger colleagues	2.41	0.80
Most older adults can stay mentally and physically fit through activities	3.55	0.59
Most older adults are disabled in their daily lives by health problems	2.22	0.73
Subjective age (1–5) ^d^	2.87	0.68
Age knowledge (0–3) ^e^	0.93	0.79
Perceived ageism (0–12)^f^	1.78	2.50

*N* = 2,000. *M* = Mean, SD = standard deviation. ^a^ Higher scores indicate higher levels of education. ^b^ Lower scores indicate better self-rated health. ^c^ Age stereotypes were recoded so that higher scores indicate endorsement of more positive age stereotypes. ^d^ Lower scores indicate a younger subjective age. ^e^ Higher scores indicate more knowledge about older adults. ^f^ Higher scores indicate more frequent experiences of ageism

## Measures

### Perceived onset of old age

Individuals were asked “At what age would you personally consider someone as old?” (perceived individual onset of old age) as well as “What do you think: At what age are people considered old in our society?” (perceived societal onset of old age), which is an item adapted from the European Social Survey (2008). We considered scores on both variables as outliers if they were more than 3 standard deviations above or below the mean (Wettstein et al. 2024); these scores were set to missing (*n* = 27 for perceived individual onset of old age and *n* = 22 for perceived societal onset of old age). As a measure of the within-person discrepancy between perceived societal onset of old age and perceived individual onset of old age, we computed an “onset of old age difference score” (perceived individual onset of old minus perceived societal onset of old age).

### Age stereotypes

Six items were used to assess age stereotypes across different domains. From the original instrument (Kornadt & Rothermund 2011) comprising eight domains, six items representing five domains were included. These items were “Most older adults can stay mentally and physically fit through activities”, “Most older adults are disabled in their daily lives by health problems” (domain physical and mental fitness, health and appearance), “Most older people are lonely” (domain family and partnership), “Most older adults have plenty of money and can spend their money for nice personal experiences” (domain financial situation and dealing with money-related issues), “Most older adults find the right solution when coping with important matters” (personality and way of living), “Most older cannot attune to changes anymore and therefore are inferior to younger colleagues” (work and employment). The response format for the items was a Likert scale ranging from 1 = fully agree to 4 = fully disagree. As each item represented a different stereotype domain and as most intercorrelations between the stereotype items were modest, ranging between 0.00 and 0.38 (only 3 correlations were > 0.20), we did not compute a composite score, but included each of the six items as an independent age stereotype. All items were transformed so that higher scores corresponded to more positive age stereotypes.

### Age knowledge

Knowledge about old age and aging was assessed based on three questions. Specifically, individuals were asked to estimate the percentage of individuals in Germany who are older than 70 years old and the percentage of individuals older than 70 years in Germany living in nursing homes. Moreover, participants were asked whether severe depressive symptoms are more common, less common, or equally common in older adults as compared to younger adults (Davis et al. 2019). For the first two questions, estimates were rated as correct if the estimated percentage was not more than 3 percentage points above or below the correct percentage (Kessler & Warner 2023). An age knowledge score was computed across all three items, ranging from 0 (no correct answer) to 3 (all three answers were correct.)

### Subjective age

Individuals were asked “What age do you feel most of the time?”, with the response options “very young,” “young,” “average,” “old,” and “very old” (Barrett 2003).

### Perceived ageism

Four items were used to assess perceived ageism (adapted from Beyer et al. 2017; Palmore 2001). Individuals were asked how often they had, within the past 2 years, experienced certain types of discrimination because of their age, such as being ignored or not taken seriously. Items were answered on a 4-point scale ranging from 1 = never to 4 = frequently. A sum score was computed (Cronbach’s alpha = 0.82), with higher scores indicating more frequent experiences of age discrimination.

### Socio-demographic indicators

We included chronological age, sex (male or female), education, and region of residence as socio-demographic indicators. Education was assessed as a variable ranging from 1 = “secondary school (or less)” to 4 = “university degree.” Study participants also indicated in which county in Germany they resided, and this information was used to derive a dichotomous region indicator (West vs. East Germany).

### Self-rated health

Self-rated health was assessed with a single-item measurement, which is a frequently used approach (DeSalvo et al. 2006; Graf & Patrick 2016). Individuals were asked “How would you rate your general health?”, with the response options ranging from 1 = very good to 5 = very bad.

## Data analysis

We computed structural equation models to estimate the associations of views on aging with perceived individual onset of old age, perceived societal onset of old age and with the onset of old age difference score. We computed separate models for individual onset of old age, perceived societal onset of old age, and the difference score in order to avoid testing too many parameters within one model (the number of estimated parameters should not get too high, and the ratio of sample size: estimated parameters should not get too small; Deng et al. 2018; Yang et al. 2018). To test for age moderation effects, we additionally computed multi-group models, specifying two age groups of similar size, namely individuals aged younger than 60 years (*n* = 1,043) and individuals aged 60 years^2^ and older (*n* = 940).

## Results

### Correlates of perceived individual onset of old age

A very good model fit was obtained when regressing perceived individual onset of old age on views on aging and the covariates (CFI = 1.00; RMSEA = 0.00, 90% CI: 0.00-0.02; SRMR = 0.007). The average perceived onset of old age was 69.92 years. Among the views on aging indicators, perceived ageism (*ß* = − 0.302, *p* = .003) and subjective age (*ß* = -1.858, *p* < .001) were significantly related to perceived onset of old age (see Table 2); individuals who reported perceived ageism more frequently and individuals who felt older set the beginning of old age earlier. In contrast, age knowledge and age stereotypes were not significantly associated with perceived individual onset of old age.

**Table 2:** Associations of views on aging and socio-demographic indicators with perceived individual and societal onset of old age. Findings from structural equation models

Predictors	Perceived individual onset of old age		Perceived societal onset of old age	
	*b* (SE)	*ß* (SE)	*b* (SE)	*ß* (SE)
Age	0.188 (0.021)***	0.409*** (0.042)	0.094*** (0.024)	0.181*** (0.047)
Women	2.941*** (0.529)	0.168*** (0.030)	1.852** (0.637)	0.094** (0.032)
East Germany	0.479 (0.652)	− 0.022 (0.030)	− 0.520 (0.774)	− 0.021 (0.031)
Education^a^	− 0.279 (0.254)	− 0.032 (0.030)	0.059 (0.277)	0.006 (0.029)
Self-rated health^b^	0.197 (0.322)	0.021 (0.034)	− 0.474 (0.412)	− 0.044 (0.038)
Age stereotypes^c^				
Most older adults find the right solution when coping with important matters	0.392 (0.373)	0.032 (0.030)	− 0.338 (0.422)	− 0.024 (0.030)
Most older people are lonely	0.702 (0.364)	0.062 (0.032)	0.774 (0.414)	0.060 (0.032)
Most older adults have plenty of money and can spend their money for nice personal experiences	− 0.525 (0.301)	− 0.049 (0.028)	− 0.115 (0.386)	− 0.009 (0.032)
Most older adults cannot attune to changes anymore and therefore are inferior to younger colleagues	− 0.214 (0.347)	− 0.020 (0.032)	− 1.073* (0.455)	− 0.088* (0.037)
Most older adults can stay mentally and physically fit through activities	0.870 (0.473)	0.060 (0.033)	0.286 (0.566)	0.018 (0.035)
Most older adults are disabled in their daily lives by health problems	0.254 (0.384)	0.021(0.032)	0.323 (0.451)	0.024 (0.033)
Subjective age^d^	− 1.858*** (0.437)	− 0.160*** (0.037)	− 0.390 (0.615)	− 0.030 (0.047)
Age knowledge ^e^	0.245 (0.351)	0.021 (0.030)	0.686 (0.404)	0.053 (0.031)
Perceived ageism ^f^	− 0.302** (0.102)	− 0.098** (0.033)	− 0.643*** (0.122)	− 0.186*** (0.035)
RMSEA (CI)	0.000 (0.00–0.02)		0.000 (0.00–0.02)	
SRMR	0.007		0.007	
CFI	1.000		1.000	
R^2^	.22		.10	

*N* = 2,000. Displayed are unstandardized (*b*) and standardized (* ß*) coefficients. *p < .05, **p < .01, *p* < .001. ^a^ Higher scores indicate higher levels of education. ^b^ Lower scores indicate better self-rated health. ^c^ Age stereotypes were recoded so that higher scores indicate endorsement of more positive age stereotypes. ^d^ Lower scores indicate a younger subjective age. ^e^ Higher scores indicate more knowledge about older adults. ^f^ Higher scores indicate more frequent experiences of ageism

**Fig. 2:**
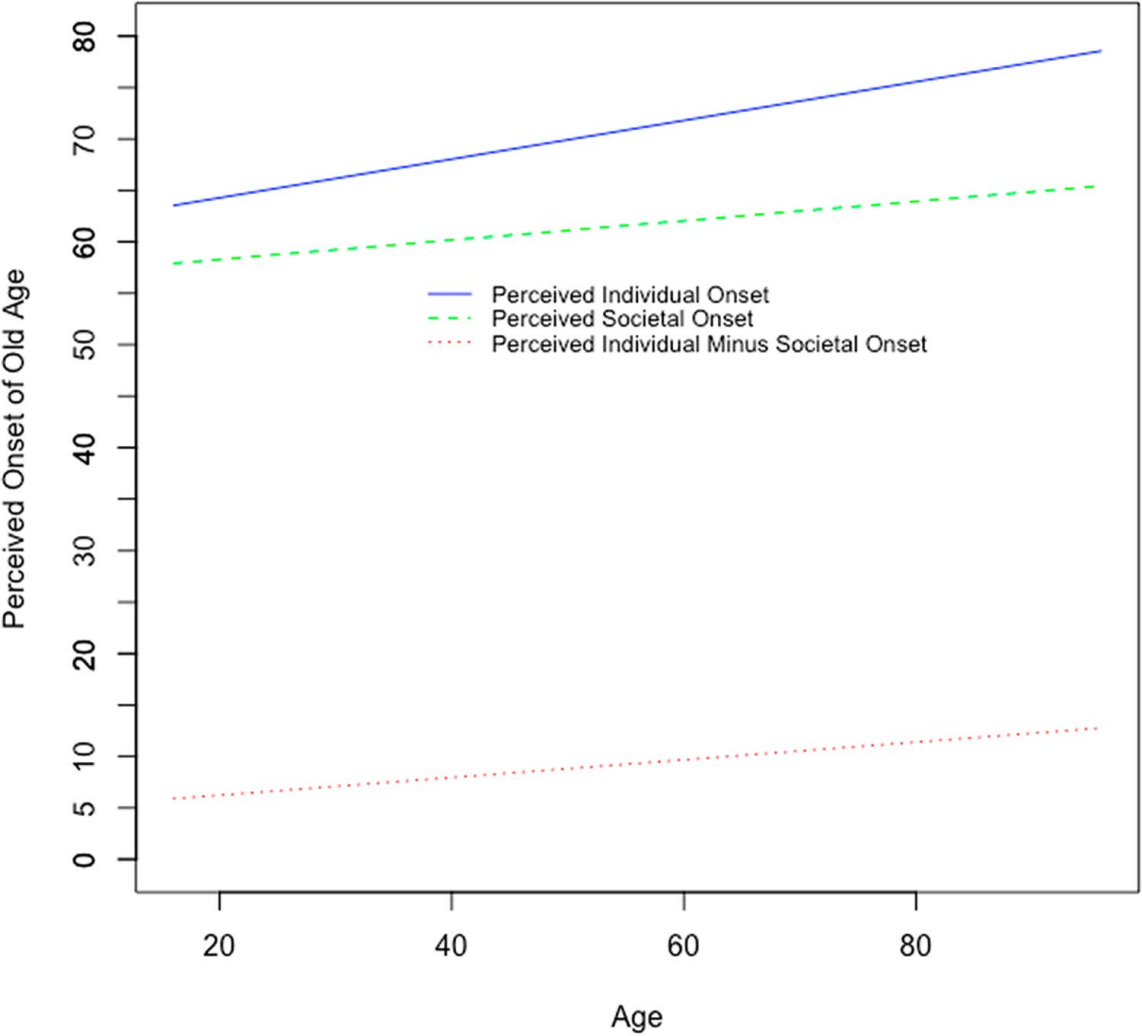
The association of chronological age with perceived individual onset of old age (blue solid line) and with perceived societal onset of old age (green dashed line). Individuals of all ages report a later perceived individual onset of old age as compared to their perceived societal onset of old age. Age differences in perceived individual onset of old age are about twice the size of age differences in perceived societal onset of old age, resulting in a discrepancy (red dotted line) between perceived individual onset of old age and perceived societal onset of old age that is more pronounced in chronologically older adults

In addition, two of the socio-demographic indicators were significantly related to the perceived individual onset of old age. An older chronological age was related to a later perceived individual onset of old age (*ß* = 0.188, *p* < .001); being chronologically older by five years was thus associated with setting the onset of old age higher by about one year (see Fig. 2). Specifically, for 16-year old persons, their predicted perceived individual onset of old age is 63.5 years, whereas for the oldest study participants aged 96 years, their estimated perceived individual onset of old age amounts to 78.6 years, corresponding to a difference of more than 15 years. Sex was also significantly related to perceived individual onset of old age (*ß* = 2.941, *p* < .001), which was on average about three years later for women as compared to men. Both the views on aging indicators and the socio-demographic variables together accounted for 22% of the interindividual variability in perceived individual onset of old age.

### Correlates of perceived societal onset of old age

Model fit was also very good when regressing the perceived societal onset of old age on views on aging as well as on socio-demographic factors (CFI = 1.00; RMSEA = 0.00, 90% CI: 0.00-0.02; SRMR = 0.007; see Table 2). The mean perceived societal onset of old age was 61.1 years, which is about nine years earlier than the perceived individual onset of old age. Individuals thus tend to set their personal beginning of old age a lot later than they think society defines the onset of old age.

Among the views on aging indicators, perceived ageism was significantly related to perceived societal onset of old age (*ß* = − 0.643, *p* < .001; see Table 2). Specifically, individuals who had experienced age discrimination more frequently set the societal onset of old age earlier than individuals with less frequent or no experiences of ageism. Moreover, one of the age stereotypes was significantly related to perceived societal onset of old age. Those individuals who more strongly disagreed with the stereotype that most older adults cannot adjust to changes reported an earlier societal onset of old age (*ß* = − 1.073, *p* = .018). The otherage stereotypes, age knowledge, and subjective age were not significantly associated with perceived societal onset of old age.

Among the socio-demographic correlates, age and sex were, in analogy with perceived individual onset of old age, also significantly associated with perceived societal onset of old age (*ß* = 0.094, *p* < .001 and *ß* = 1.852, *p* = .004). Being 10 years chronologically older was associated with setting the perceived societal onset of old age about one year later; the age gradient for perceived societal onset of old age was thus less steep than the age gradient for perceived individual onset of old age (see Fig. 2). The estimated perceived societal onset of old age is 57.9 years for individuals aged 16 years, whereas for individuals aged 96 years, the estimated perceived societal onset is 65.4 years, thus more than 8 years later. Women’s perceived societal onset of old age was almost 2 years later compared to men. Altogether correlates including views on aging and socio-demographic indicators explained 10% of interindividual variation in perceived societal onset of old age.

### Correlates of the difference between perceived individual onset of old age and perceived societal onset of old age

A very good model fit also resulted from the model with the discrepancy between perceived societal onset of old age and perceived individual onset of old age as outcome (CFI = 1.00; RMSEA = 0.000, 90% CI: 0.00-0.02; SRMR = 0.007; see Table 3). The discrepancy, pointing toward a later perceived societal onset of old age as compared to the perceived individual onset of old age, was greater by 1.6 years among women as compared to men. It was also greater among chronologically older persons (see Fig. 2) as well as among persons who reported having experienced ageism. None of the other views on aging indicators and socio-demographic factors were significantly associated with the onset of old-age difference score. A total of 5% of the interindividual variability in the difference score was explained by the included predictors.

**Table 3:** Associations of views on aging and socio-demographic indicators with the difference of perceived individual and societal onset of old age. Findings from structural equation models

Predictors	Perceived individual onset of old age minus Perceived societal onset of old age	
	*b* (SE)	*ß* (SE)
Age	0.086*** (0.024)	0.171*** (0.047)
Women	1.643** (0.630)	0.086** (0.033)
East Germany	0.569 (0.749)	0.024 (0.031)
Education^a^	− 0.325 (0.290)	− 0.034 (0.031)
Self-rated health^b^	0.106 (0.342)	0.010 (0.033)
Age stereotypes^c^		
Most older adults find the right solution when coping with important matters	0.740 (0.440)	0.054 (0.032)
Most older people are lonely	− 0.115 (0.415)	− 0.009 (0.033)
Most older adults have plenty of money and can spend their money for nice personal experiences	− 0.341 (0.356)	− 0.029 (0.030)
Most older adults cannot attune to changes anymore and therefore are inferior to younger colleagues	0.623 (0.457)	0.052 (0.039)
Most older adults can stay mentally and physically fit through activities	0.740 (0.600)	0.047 (0.038)
Most older adults are disabled in their daily lives by health problems	− 0.056 (0.453)	− 0.004 (0.034)
Subjective age^d^	− 0.947 (0.637)	− 0.074 (0.049)
Age knowledge ^e^	− 0.447 (0.390)	− 0.035 (0.031)
Perceived ageism ^f^	0.291* ( 0.117)	0.087* (0.035)
RMSEA (CI)	0.000 (0.00–0.02)	
SRMR	0.007	
CFI	1.000	
R^2^	.05	

*N* = 2,000. Displayed are unstandardized (*b*) and standardized (*ß*) coefficients. *p < .05, **p < .01, *p* < .001. ^a^ Higher scores indicate higher levels of education. ^b^ Lower scores indicate better self-rated health. ^c^ Age stereotypes were recoded so that higher scores indicate endorsement of more positive age stereotypes. ^d^ Lower scores indicate a younger subjective age. ^e^ Higher scores indicate more knowledge about older adults. ^f^ Higher scores indicate more frequent experiences of ageism

## The moderating role of chronological age

We compared individuals younger than 60 years (*n* = 1,043) with individuals aged 60 years and older (*n* = 940); the model fit of a model that specified associations of perceived individual onset of old age with views on aging and socio-demographic variables as varying across both age groups was not significantly different from the model fit of a model with these associations constrained to be equal across groups (Satorra–Bentler chi-square difference; Δ*χ*2 (14) = 21.57, *p* = 0.09).^3^ Associations can thus be assumed equal across both age groups. For perceived societal onset of old age, the Satorra–Bentler chi-square difference test was significant (Δ*χ*2 (14) = 71.51, *p* < .001; model fit of the resulting model: RMSEA = .000, CFI = 1.000, SRMR = .009), indicating that associations of perceived societal onset of old age with views on aging and socio-demographic indicators varied across age groups. Specifically, whereas perceived ageism was significantly associated with an earlier perceived societal onset of old age in both age groups (*ß* = − 0.747, *p* < .001, and *ß* = − 0.755, *p* < .001; see Table 4), different stereotypes were associated with perceived societal onset of old age in both groups. In the younger group, individuals who disagreed more with the age stereotype that older adults can no longer adjust to changes set the societal onset of old age earlier as compared to individuals who tended to agree to that age stereotype (*ß* = − 1.216, *p* = .049). In the older age group, persons who reported more agreement with the statement that “most older adults find the right solution when coping with important matters” set the perceived societal onset of old age earlier than those who disagreed with this age stereotype (*ß* = − 1.427, *p* = .018). Moreover, socio-demographic variables differed between age groups; only in the younger age group, women set the societal onset of old age later than men, with a difference of about 2 ½ years (*ß* = 2.544, *p* = .003). Only in the older group, being chronologically older was significantly associated with a later perceived societal onset of old age (*ß* = 0.299, *p* < .001); specifically, being three years older was associated with a later perceived societal onset of old age of about one year.

**Table 4:** Associations of views on aging and socio-demographic indicators with perceived societal onset of old age in adults aged 16–59 years and in adults aged 60 years and older. Findings from multi-group structural equation models

Predictors	Group 16–59 years		Group 60 years and older	
	*b* (SE)	*ß* (SE)	*b* (SE)	*ß* (SE)
Age	0.026 (0.147)	0.034 (0.056)	0.299*** (0.054)	0.262*** (0.046)
Women	2.544** (0.868)	0.131** (0.045)	0.717 (0.779)	0.037 (0.040)
East Germany	− 0.118 (1.042)	− 0.005 (0.042)	− 1.055 (1.043)	− 0.045 (0.044)
Education^a^	− 0.133 (0.371)	− 0.013 (0.038)	0.476 (0.350)	0.050 (0.036)
Self-rated health^b^	− 0.583 (0.551)	− 0.056 (0.053)	0.348 (0.496)	0.030 (0.043)
Age stereotypes^c^				
Most older adults find the right solution when coping with important matters	0.074 (0.530)	0.005 (0.038)	− 1.427* (0.604)	− 0.104 * (0.043)
Most older people are lonely	0.728 (0.605)	0.055 (0.046)	0.381 (0.511)	0.032 (0.043)
Most older adults have plenty of money and can spend their money for nice personal experiences	− 0.174 (0.524)	− 0.014 (0.043)	0.117 (0.542)	0.010 (0.046)
Most older adults cannot attune to changes anymore and therefore are inferior to younger colleagues	− 1.216* (0.618)	− 0.099* (0.050)	− 0.470 (0.552)	− 0.040 (0.047)
Most older adults can stay mentally and physically fit through activities	0.448 (0.776)	0.028 (0.049)	0.349 (0.624)	0.021 (0.038)
Most older adults are disabled in their daily lives by health problems	0.578 (0.575)	0.043 (0.043)	0.349 (0.624)	0.055 (0.053)
Subjective Age^d^	− 0.800 (0.795)	− 0.059 (0.060)	0.906 (0.744)	0.061 (0.050)
Age knowledge ^e^	0.678 (0.540)	0.053 (0.042)	0.729 (0.518)	0.057 (0.040)
Perceived ageism ^f^	− 0.747*** (0.147)	− 0.234*** (0.045)	− 0.755*** (0.197)	− 0.180*** (0.047)
R^2^		.098		.132

*N* = 2,000. Displayed are unstandardized (*b*) and standardized (* ß*) coefficients. *p < .05, **p < .01, *p* < .001. ^a^ Higher scores indicate higher levels of education. ^b^ Lower scores indicate better self-rated health. ^c^ Age stereotypes were recoded so that higher scores indicate endorsement of more positive age stereotypes. ^d^ Lower scores indicate a younger subjective age. ^e^ Higher scores indicate more knowledge about older adults. ^f^ Higher scores indicate more frequent experiences of ageism

The Satorra–Bentler chi-square difference test was also statistically significant for discrepancy between perceived individual and perceived societal onset of old age (Δ*χ*2 (14) = 67.11, *p* < .001; model fit of the resulting model: RMSEA = .000, CFI = 1.000, SRMR = .009). While perceived ageism was related to a larger discrepancy in both the younger and the older group, an older chronological age was related to a larger discrepancy in the younger group and with a smaller discrepancy in the older group (see Table 5). Moreover, only in the older group, the discrepancy was larger for women, for those feeling younger, and for those endorsing more positive age stereotypes regarding personality and way of living.

**Table 5:** Associations of views on aging and socio-demographic indicators with the difference between perceived individual onset of old age and perceived societal onset of old age in adults aged 16–59 years and in adults aged 60 years and older. Findings from multi-group structural equation models

Predictors	Group 16–59 years		Group 60 years and older	
	*b* (SE)	* ß* (SE)	*b* (SE)	* ß* (SE)
Age	0.160*** (0.040)	0.214*** (0.052)	− 0.131** (0.049)	− 0.113** (0.043)
Women	1.556 (0.826)	0.083 (0.044)	2.056* (0.798)	0.105* (0.040)
East Germany	1.258 (1.029)	0.052 (0.042)	− 0.353 (0.906)	− 0.015 (0.038)
Education^a^	− 0.476 (0.382)	− 0.050 (0.040)	− 0.164 (0.360)	− 0.017 (0.037)
Self-Rated Health^b^	− 0.074 (0.416)	− 0.007 (0.041)	− 0.647 (0.495)	− 0.056 (0.042)
Age Stereotypes^c^				
Most older adults find the right solution when coping with important matters	0.500 (0.549)	0.038 (0.041)	1.804** (0.592)	0.130 (0.042)
Most older people are lonely	0.483 (0.592)	0.038 (0.047)	− 0.259 (0.548)	− 0.022 (0.045)
Most older adults have plenty of money and can spend their money for nice personal experiences	− 0.088 (0.471)	− 0.008 (0.040)	− 0.720 (0.507)	− 0.061 (0.042)
Most older adults cannot attune to changes anymore and therefore are inferior to younger colleagues	0.579 (0.608)	0.049 (0.052)	− 0.010 (0.523)	− 0.001 (0.045)
Most older adults can stay da and physically fit through activities	0.247 (0.780)	0.016 (0.052)	0.857 (0.717)	0.051 (0.042)
Most older adults are disabled in their daily lives by health problems	− 0.045 (0.597)	− 0.003 (0.046)	− 1.223 (0.664)	− 0.093 (0.050)
Subjective age^d^	− 0.194 (0.777)	− 0.015 (0.060)	− 3.156*** (0.743)	− 0.209**(0.049)
Age knowledge^e^	− 0.968 (0.500)	− 0.079 (0.041)	0.708 (0.556)	0.054 (0.043)
Perceived ageism ^f^	0.336* (0.133)	0.109* (0.044)	0.695** (0.204)	0.164*** (0.047)
R^2^		.067		.142

*N* = 2,000. Displayed are unstandardized (*b*) and standardized (*ß*) coefficients. * *p* < .05, ** *p* < .01, *p* < .001. ^a^ Higher scores indicate higher levels of education. ^b^ Lower scores indicate better self-rated health. ^c^ Age stereotypes were recoded so that higher scores indicate endorsement of more positive age stereotypes. ^d^ Lower scores indicate a younger subjective age. ^e^ Higher scores indicate more knowledge about older adults. ^f^ Higher scores indicate more frequent experiences of ageism

## Discussion

In this study, we built on the theoretical framework by Diehl and Wahl (2024) and investigated how perceived individual and societal onset of old age as well as their discrepancy are related to socio-demographic factors, self-rated health, and views on aging in a large, representative, and age-heterogeneous sample of adults in Germany. Moreover, we investigated whether these associations are moderated by chronological age.

### Views on aging and perceived onset of old age

Associations of views on aging with perceived onset of old age varied according to the specific views on aging indicator considered, which is in line with a multidimensional conception of views on aging (Kornadt et al. 2020; Shrira et al. 2022). Moreover, our findings suggest that perceived individual onset of old age and perceived societal onset of old age do not represent the same construct as associations with views on aging varied considerably for both outcomes.

We were able to identify similarities and differences in how various views on aging indicators were related to both onset of old age variables and their discrepancy. With regard to the similarities, more frequent experiences of ageism were related to a perceived earlier individual and societal onset of old age; notably, this association was more than twice as strong for perceived societal onset of old age as compared to perceived individual onset of old age. This stronger association of perceived ageism with societal onset of old age might also explain why the discrepancy of perceived societal and individual onset of old age was greater among individuals with experienced ageism. From their experienced—potentially societal—ageism, participants may have inferred that society sets an earlier onset of old age, resulting in a larger discrepancy between perceived societal onset of old age and perceived individual onset of old age; but interestingly, they also personally set an earlier onset of old age when affected by more frequent experiences of ageism, which may reflect an “embodiment” (Levy 2009) of ageism or a “contamination effect”. However, given the cross-sectional study design, we cannot rule out the possibility that the causal pathway is reversed, and that individuals who perceive on earlier onset of old age, thus potentially considering themselves as “old”, may be more likely to attribute negative experiences or unpleasant social interactions to ageism. In contrast, for individuals with a later perceived onset of old age, being old may be “far away” and these individuals may therefore not attribute similar negative experiences to discrimination due to age.

Regarding differences in how views on aging are related to perceived individual and societal onset of old age, subjective age was only significantly related to perceived individual onset of old age. Consistent with other findings (Toothman & Barrett 2011; Wettstein et al. 2024), individuals who feel younger set the individual onset of old age later, whereas how old individuals feel does not seem to be relevant to when they think society considers a person as old. Feeling younger may affect one’s individual conceptions of age, and someone who reports feeling “young” old at age 60 will probably believe that old age does by no means start as early as at age 60. However, it could again be the opposite causal pathway, with individuals who believe, for instance, that old age starts at age 60 will feel “old” as soon as they reach that age. Further research is therefore needed to investigate how subjective age and perceived onset of old age precede and predict each other longitudinally over time.

Whereas subjective age, which is an indicator of personal views on aging (Shrira et al. 2022), was only related to the perceived individual onset of old age, age stereotypes, which reflect generalized views on aging and refer to older adults in society, were only significantly related to perceived societal onset of old age. However, this was true only for one out of the six stereotype domains, namely work and employment. Thus, it seems that only certain age stereotypes are associated with perceived societal onset of old age. Specifically, individuals who believe that most older adults can no longer adjust to changes and are therefore inferior to younger adults set the societal onset of old age later than individuals who disagreed with this stereotype. For the perceived onset of old age, the aspect of “work” might be particularly salient. For instance, Augustyński and Jurek (2021) found that the average perceived onset of old age was later in countries with higher employment rates and labor force participation of older adults. This particular relevance of work-related aspects might explain why only this stereotype domain was significantly related to the perceived onset of old age. Endorsing positive age stereotypes could mean that old age is not seen as an undesirable state, so that people do not feel pressured to disidentify themselves from the group of older adults by setting the old-age threshold further away from them. However, the reversed causality is also plausible, in that perceived societal onset of old age shapes age stereotypes: Those who think that early-old age is the beginning of old age may tend to disagree to the stereotype that older adults cannot adjust to changes, whereas those for whom old age starts in fourth age (Baltes & Smith 2003; Wahl & Ehni 2020), that is at age 80 or later , may be more likely to agree with such a stereotype.

It is interesting that the age stereotype related to work and employment was associated only with the perceived societal onset of old age. As age stereotypes are “non-self-referential” (Faudzi et al. 2019) and correspond to general views on aging (Shrira et al. 2022), they might not be immediately relevant for the perceived individual onset of old age, but for the perceived societal onset of old age—which can also be regarded as “non-self-referential”. In contrast, views on aging indicators that are self-referential and represent personal views on aging (Shrira et al. 2022), such as subjective age, are—according to our findings—significantly related to the perceived individual onset of old age, but not with the perceived societal onset of old age.

### Associations of socio-demographic factors with individual versus societal onset of old age

Similarities, rather than differences were found between the two onset of old-age indicators regarding their associations with socio-demographic indicators. Specifically, being female and being chronologically older were both associated with setting both onsets of old age at a later age, which is in line with prior research (Ayalon et al. 2014; Chopik et al. 2018; Rupprecht et al. 2025; Wettstein et al. 2024; Wurm et al. 2025). Women also perceive a particularly large gap between the age they consider as old and the age society considers as old. As discussed above, women live longer than men (Eurostat 2022; German Federal Statistical Office, n.d.), feel younger than men (Wettstein et al. 2023), and older women are typically portrayed more negatively than older men in the media (Bazzini et al. 1997; Lauzen 2021; Lauzen & Dozier 2005), which might lead to a stronger tendency of women as compared to men to employ “age-group dissociation” by setting the onset of old age later. Another explanation could be that women are aware that society considers them to transition into old age at an earlier age than men (Barrett & Von Rohr 2008; Billari et al. 2021; Drevenstedt 1976; Toothman & Barrett 2011), whereas at the same time, women perceive old age to start later than men (Ayalon et al. 2014; Barrett & Von Rohr 2008; Chopik et al. 2018; Drevenstedt 1976; Frąckowiak et al. 2020; Toothman & Barrett 2011).

Being chronologically older by five years was associated with setting the perceived individual onset of old age higher by about one year and with setting the perceived societal onset of old age higher by about 0.5 years. The age gradient for perceived societal onset of old age was thus less steep than the age gradient for the perceived individual onset of old age, resulting also in a greater deviation between perceived individual onset of old age and perceived societal onset of old age with advancing age (see Fig. 2). Chronologically older adults may feel particularly urged to employ “age-group dissociation” and to set the old-age threshold higher in order to still remain below that threshold, whereas they believe that society has a lower old-age threshold.

Interestingly, mean perceived individual onset of old age vs. mean societal onset of old age were about nine years apart (Kessler & Warner 2023). Most people thus believe that society considers someone as old much earlier than they personally consider someone as old. Participants may have had the statutory retirement age in Germany in mind when specifying the societal onset of old age (which is between 65 and 67 years, depending on birth cohort). In 2022, the average age when transitioning to retirement in Germany was 64.4 years (Demografieportal 2025). When reporting their perceived societal onset of old age, participants may also have been aware that a considerable proportion of middle-aged and older adults report being affected by age discrimination (Beyer et al. 2017), particularly in the work domain. With this in mind, participants may have concluded that society considers people to be old at an earlier age than they personally do. In contrast, individuals’ personal onset of old age is much later. This could be due to an “age-group dissociation” effect (Weiss & Freund 2012; Weiss & Kornadt 2018; Weiss & Lang 2012): As most people do not want to be old, they “postpone” their perceived onset of old age to distance themselves from the group of older adults. However, individuals might also have certain “role models” in their personal environment who are older adults but whom they would not consider as old and therefore report a later individual onset of old age as compared to their perceived societal onset of old age.

### The role of chronological age as a moderator between views on aging and perceived onset of old age

No differences in the associations of perceived individual onset of old age with views on aging and socio-demographic indicators were found when comparing individuals aged under 60 years with those aged 60 years and over. Also, when we used an alternative age-group categorization and contrasted those younger than 65 with those aged 65 years and older, associations were not significantly different between age groups. These associations therefore seem to some extent “age-invariant.” Nevertheless, we acknowledge that our cutoff point of 60 years (and also the alternative cutoff of 65 years) was somewhat arbitrary. In aging research, the beginning of old age is often set at this cutoff point of 60 years, and another advantage of this age-group categorization was that the resulting subgroups were similar in size. However, it would have been desirable to distinguish more groups representing different life phases, ideally young adulthood (20–40 years), midlife (40–60 years; Lachman 2015), early-old age and very old age (60–80 years vs. 80 years and older; Baltes & Smith 2003; Wahl & Ehni 2020), but such an approach would have violated the principle of model parsimony, with too many group-specific parameters to be estimated, and would have required a larger total sample size. With our comparison of only two groups, we could at least make sure that the sample sizes of both subgroups are comparable, which would not have been possible when comparing the four life stages mentioned above. However, future research should investigate whether correlates of perceived individual onset of old age vary with chronological age by considering more than two age groups and also by taking into account the potential role of age as a nonlinear moderator.

When investigating the role of age for the perceived societal onset of old age and for the discrepancy between perceived individual and societal onset of old age, several age-specific findings emerged. Specifically, while perceived ageism was significantly related to an earlier perceived societal onset and to a greater difference between perceived individual and perceived societal onset of old age in both age groups, underlining the universal role of ageism, different age stereotypes were associated with perceived societal onset of old age in both age groups. Specifically, in the younger group, endorsing positive age stereotypes regarding work and employment was related to an earlier perceived societal onset of old age, potentially due to the reasons and mechanisms we discussed above. These age stereotypes were not a significant factor in the older group, with most of them being retired so that age stereotypes related to work might simply be less salient and less relevant for them. For older adults, however, stereotypes related to personality and way of living seem to gain in importance, as those endorsing more positive stereotypes in this domain reported an earlier perceived onset of old age, whereas this stereotype domain was not significantly related to the perceived societal onset of old age in the younger age group. 

While these findings require replication, one potential conclusion is that the association of age stereotypes with perceived onset of old age depends on (1) whether perceived individual vs. perceived societal onset of old age (or the difference between both measures) is considered, (2) the specific stereotype domain and content, and (3) the specific age group that is considered.

## Study limitations

There are several study limitations that need to be pointed out. Specifically, as mentioned above, given the cross-sectional study design, causal conclusions are not warranted, and views on aging could be either predictors of perceived onset of old age or outcomes, or both predictors and outcomes. Due to the cross-sectional study design, we can also not rule out that age differences in perceived onset of old age reflect or are confounded with cohort effects, and prior research has indeed shown that perceived onset of old age is subject to historical change (Augustyński & Jurek 2021; Ennis et al. 2025; Wettstein et al. 2024).

It is also important to point out that our findings from a German sample may not be generalizable to other countries, given that countries—even within Europe—are quite different regarding factors associated with the perceived onset of old age, such as older adults’ employment rate and mandatory retirement ages. It is thus not surprising that remarkable differences in the perceived onset of old age between various European countries were observed (Augustyński & Jurek 2021; Ayalon et al. 2014; Swift et al. 2022).

We assessed when people consider “someone” as old, and this in line with how prior research assessed the personal onset of old age. However, future studies could also consider including the question “When do you personally consider yourself as old?”.

Another limitation related to our measures is that some of the assessment instruments were quite short, in order to reduce the burden for participants. For instance, our 3-item measure of age knowledge should be regarded as a “screening tool”, but for a more accurate assessment of general knowledge about the characteristics and life circumstances of older adults, longer instruments—such as an updated version of Palmore’s Facts on Aging Quiz (Palmore 1988)—may be more appropriate. Also, subjective age was assessed based on one 5-point Likert response format. While this item was also used in prior research (e.g., Barrett 2003), a different operationalization of subjective age with more than five response categories (such as subjective age proportional discrepancy; Pinquart & Wahl 2021; Rubin & Berntsen 2006) might have been more accurate and would potentially have resulted in stronger associations between subjective age and perceived onset of old age. Also, there is no direct comparability between the 5-point response format to asses subjective age used in this study and studies which measured “numerical” subjective age and computed the subjective age proportional discrepancy.

We were not able to include objective indicators, e.g., of health, as they were not assessed as part of the telephone-based survey. Also, as noted above, our age-group comparison was limited to only two age groups, so that we might have missed potential nonlinear age moderation effects, which requires further research.

Finally, views on aging comprise both personal and general views on aging (Shrira et al. 2022). However, subjective age was the only available personal views on aging indicator, and there may be other indicators, such as attitude toward one’s own aging (Lawton 1975) or awareness of age-related change (Diehl et al. 2014; Diehl & Wahl 2010), which could be meaningfully related to perceived onset of old age.

## Conclusion

In this study, we investigated perceived individual and societal onset of old age in an age-heterogeneous sample and found that, on average, individuals set their personal onset of old age more than eight years later than their perception of when society considers someone as old, and this discrepancy was particularly pronounced among those with experienced ageism, among women, and among chronologically older individuals. Similarities and differences were observed when investigating how views on aging were related to both onsets of old age. Specifically, perceived ageism was significantly related to both an earlier perceived individual and societal onset of old age, whereas positive age stereotypes were only related to an earlier perceived societal onset of old age, and feeling younger was associated only with a later perceived individual onset of old age. Thus, our findings suggest that determinants or correlates of perceived individual vs. societal onset of old age are not the same, and that subjective experiences of aging are differentially related to both onset of old age indicators. Further research is needed to investigate the mechanisms underlying associations between perceived onset of old age and views on aging.

## Data Availability

Data usage requests can be addressed to the Federal Anti-Discrimination Agency of Germany (poststelle@ads.bund.de).

## Footnotes

1 Representativeness was ensured by including sampling weights in the analyses. In order to obtain a representative study sample, different selection probabilities (due to a different number of landline or mobile phone numbers and target persons living in the household) were balanced by design weighting. The structural weighting corrected for non-response-related selectivity in the sample. The weighting factors were based on the comparison of demographic characteristics of the sample with the population structure according to Federal Statistical Office (for distribution by gender and age groups) and the current German microcensus (for the characteristic of school leaving qualifications). The weighting included the factors age, education, region, and household size.

2 The cutoff at age 60 years was not only chosen for the sake of obtaining two equally large groups, but also because “from ancient literature to contemporary times, most authorities have located the beginning of old age around age sixty” (Covey 1992; p. 325).

3 The age of 65 years might be an alternative cutoff; as by age 65, most individuals in Germany are retired (Demografieportal 2025). We therefore repeated the multigroup analyses by differentiation between those younger than 65 years (*n* = 1,338) and those aged 65 years and older (*n* = 645). Again, the Satorra–Bentler chi-square difference was not significant (Δχ^2^ (14) = 20.06, *p* = .22), indicating that associations of views on aging and socio-demographic factors with perceived individual onset of old age were not significantly different between the younger and the older group.
